# Preparing for Cancer: A Qualitative Study of Hispanic Patient and Caregiver Needs

**DOI:** 10.3390/healthcare12111117

**Published:** 2024-05-30

**Authors:** Lianel Rosario-Ramos, Stephanie Torres-Marrero, Tiffany Rivera, Maria Elena Navedo, Rosael Burgos, Mayra Garriga, Maria del Carmen Pacheco, Betsy Lopez, Yamilet Torres, Normarie Torres-Blasco

**Affiliations:** 1 School of Behavioral and Brain Sciences, Ponce Health Sciences University, Ponce 00716, Puerto Rico; lrosario21@stu.psm.edu (L.R.-R.); storres21@stu.psm.edu (S.T.-M.); tirivera21@stu.psm.edu (T.R.); rburgos20@stu.psm.edu (R.B.); mgarriga20@stu.psm.edu (M.G.); mpacheco20@stu.psm.edu (M.d.C.P.); belopez20@stu.psm.edu (B.L.); ytorres20@stu.psm.edu (Y.T.); 2 Department of Medicine, University of Connecticut, Storrs, CT 06269, USA; menavedomd@gmail.com; 3 Ponce Research Institute, Ponce 00716, Puerto Rico

**Keywords:** cancer, Hispanic, dyads, patients, caregivers, providers, preparedness needs, psychosocial interventions

## Abstract

Background: Cancer disproportionately affects Hispanic populations, yet the preparedness of Hispanic caregiver–patient dyads facing cancer remains understudied. This study aims to identify essential components of preparedness needs and inform future psychosocial interventions for this demographic. Methods: Secondary analyses were conducted utilizing focus groups to develop a communication intervention for Hispanic patients and caregivers. Transcripts were qualitatively analyzed using NVivo v12 (2020). Results: Analysis revealed symptom management and treatment comprehension as pivotal aspects of preparation. Additionally, preparedness among our sample emerged by addressing the multifaceted dimensions of preparedness, including psychological, emotional, educational, familial, practical, financial, and spiritual aspects. Conclusions: Tailoring interventions encompassing diverse dimensions of preparedness can foster inclusivity and maximize their impact on supportive measures. This underscores the necessity for culturally sensitive approaches when delivering interventions supporting Hispanic individuals navigating the challenges of cancer.

## 1. Introduction

Cancer is a leading cause of mortality among Hispanics in both the continental United States and Latin American countries [1]. This population is often diagnosed with cancer at an advanced stage, which impacts the physical, mental, financial, spiritual, and social domains [2]. When combined, these factors can lead to significant distress and interfere with a patient’s ability to cope effectively with their cancer diagnosis and subsequent care [3].

Cancer care encompasses the extent to which patients receive comprehensive and personalized treatment, support, and resources to manage their condition and improve their quality of life [4,5]. This preparedness extends to planning and readiness for the emotional and practical aspects of dealing with a cancer diagnosis, treatment, and journey, as well as its impact on the patient and their caregivers [6,7]. Preparedness goes beyond prognosis awareness or acceptance; it involves completing tasks, processes, coordination, and actions deemed necessary by the patient, ultimately leading to acceptance [8,9,10]. This active approach is reflected in how patients with cancer define preparedness.

Patients with cancer define preparedness as encompassing planning, effective communication, symptom management, acceptance or avoidance of prognosis, working on relationships with loved ones, emotional well-being, spiritual comfort, discussing finances, and preparation for death [6]. Moreover, autonomy and empowerment relate to patients when setting and achieving goals and decisions for themselves [11]. Nevertheless, it is crucial to recognize that patients’ preferences regarding personal, emotional, social, spiritual, and financial factors may vary [12,13,14]. Healthcare providers also play an essential role in the acceptance process and preparedness by providing information about the illness, treatment options, and overall prognosis [15,16,17]. Patients who feel adequately prepared after a cancer diagnosis and are involved in decision-making regarding their care tend to experience an improved quality of life [18,19,20].

The need for preparedness covers patients and extends to caregivers, usually family members, who aid patients on their cancer journey and can significantly improve their well-being [7,21,22]. However, caregivers often report feeling unprepared to navigate various cancer-related aspects, including accessing resources, understanding healthcare services, cancer-related information, treatment, and prognosis [23,24,25,26,27]. Caregiver preparedness also includes providing emotional support, fostering effective communication among patients, caregivers, and healthcare providers, and educating themselves about the entire process [7]. There are also practical decisions for the caregiver that can be burdensome, such as organizing legal documents, managing financial affairs, and an assortment of arrangements [28,29]. Their sense of preparedness is associated with their ability to adapt successfully to the experience, influencing their decision-making regarding patient needs, medical interventions, and patient support [30,31,32].

Both patients and caregivers may find themselves unprepared to face these challenges, providing a gap in the impact on the quality of life for caregiver–patient dyads. Specifically, among Hispanic patients with cancer, the preparedness of patient–caregiver dyads remains insufficiently studied. To address these needs and provide possible treatment, preparedness components must be understood. Thus, the overall objective of this study is to identify and present the essential components of preparedness needs that are crucial for Hispanic caregiver-patient dyads, as well as community partners and healthcare providers. By considering a broader range of individuals involved in the care process, this study aims to inform and enhance future psychosocial supportive interventions to assist in preparedness.

## 2. Materials and Methods

This study is a secondary analysis focusing on the preparedness needs of patient–caregiver dyads. Building on the findings of the parent study, this analysis aims to explore the broader spectrum of preparedness, encompassing not just the needs of the patients and their caregivers but also the roles of community partners and healthcare providers.

### 2.1. Parent Study

The parent study aimed to develop a community-based communication intervention for Latino patients and caregivers coping with cancer [33]. Thus, the principal study was guided by community engagement research (CER), which allows direct access to populations alongside laypersons who are familiar with the community or may have experience with the research topic [34,35]. For this study, an academic researcher (NTB) collaborated with four community researchers (RB, MG, BL, and CP), who are community partners in rural towns in southern Puerto Rico (Villalba, Lajas, Peñuelas, and Yauco) with personal experiences related to cancer.

The participants were recruited with the support of the community partners (RB, MG, BL, and CP), who obtained community-based participatory research training. This ensures effective participant recruitment and engagement, allowing community partners to actively participate in the research process, from setting research priorities to disseminating findings. This study enrolled 27 participants aged 21 years and older, including eight patients with cancer, eight caregivers, seven community partners, and four healthcare providers. The initial study conducted focus groups, allowing the investigation of collective perspectives, attitudes, behaviors, and experiences, facilitating the acquisition of rich, in-depth data, and uncovering consensus and disparities within and between groups. The parent study provides more detailed information regarding its methodology and the specific questions used to guide these focus groups, which were designed to elicit comprehensive insights into the participants’ needs and experiences [36]. In the focus groups, a semi-structured interview was conducted, which included questions regarding developing a communication intervention and critical needs among patients. Afterward, the research team transcribed the focus groups.

### 2.2. Secondary Analysis

This study is a secondary analysis of the original transcript of the initial parent study. The analyses yielded additional needs not related to communication. Regarding it, preparedness needs and the need to address them arose from the focus groups.

#### Data Analysis

For this secondary analysis, the focus group transcripts were analyzed by a qualitative thematic content analysis utilizing NVivo v12 (2020). Qualitative thematic content analysis allows investigators to identify, analyze, and report patterns within the data corpus, regardless of whether that theme captures most experiences [37]. During the initial analysis, the primary author read the focus group transcripts and identified the most salient topic reported by the participants related to preparedness needs. The primary author (L.R.-R.) developed the codes and themes and defined them. After developing the themes/codes, qualitative analysts (L.R.-R., S.T.M., and T.R.) independently coded the transcripts. Iterative discussions among analysts ensured consensus on coding interpretations, enhancing the rigor and reliability of the analysis. Finally, a comprehensive coding dictionary was established through continuous discussions, facilitating consistent coding across all transcripts.

## 3. Results

This study involved 27 participants who, on average, were 53 years old. Participants’ sociodemographic characteristics described that the majority of the participants were female and were married. Furthermore, results indicated that participants reported an average income of USD 25,880. Concerning participant characteristics, 30% represented patients and caregivers, 26% were community partners, and 15% were healthcare providers. See Table 1.

### 3.1. Qualitative Themes on Preparedness

Preparedness refers to the active planning and readiness for various aspects of a diagnosis and treatment and its impacts on the patient and their caregivers. This involves both practical and emotional preparation to navigate the challenges that cancer can present. Through focus group discussions, participants shed light on various facets of preparedness, ranging from psychological and educational needs to the practical, financial, and spiritual considerations of the cancer journey. For this reason, preparedness needs aroused the themes of psychological and emotional, education, inclusion of the family, symptoms and treatment, healthcare provider, practical and care, economic, and spiritual. 

### 3.2. Psychological and Emotional Preparedness Needs

When participants mentioned the need or desire to gain preparedness related to psychological or emotional aspects, they expressed the necessity of addressing the mental and emotional challenges associated with cancer diagnosis, treatment, and caregiving. Two patients emphasized the importance of acknowledging caregivers’ emotional needs, stating, “(We are concerned about) The patient. But what about the caregiver? They weep, too” (P07, female patient; P10, female patient).

Similarly, a caregiver stressed the value of allowing patients to express their emotions freely before offering encouragement, stating, “Before delivering words of encouragement, it is best to inquire, ‘How do you feel?’ Make space for the patient to express their feelings and vent. This may be an essential part of the intervention” (P18, male caregiver). Additionally, a community partner emphasized the necessity for emotional and psychological preparedness for caregivers, recognizing the draining nature of caregiving, stating, “The caregiver also needs to prepare emotionally and psychologically, as the process can be very draining” (P23, female community partner).

### 3.3. Education Preparedness Needs

Education emerged as another critical aspect of preparedness. Participants expressed a desire for comprehensive education tailored to individual needs. One patient emphasized the importance of respecting the uniqueness of each cancer experience, stating, “Not all cases are the same. I can provide you with information, but it’s important to remember that, this will happen to you. Not everything is accurate because each case, the symptoms, and the environment are different” (P19, female patient).

Similarly, a caregiver highlighted the need for guidance and support for caregivers and family members, stating, “For me, it is about guiding everyone involved—the patients, the caregivers, and their family members—through everything that comes with cancer. This includes preparing them for the ups and downs and helping to strengthen both the caregiver and the patient” (P12, female caregiver).

### 3.4. Inclusion of Family Preparedness Needs

The theme of including family members who are not caregivers emerged as a significant consideration. Participants emphasized the importance of involving extended family members in the caregiving process for support. A patient highlighted the necessity of including family members, even children, as immediate support networks, stating, “We can’t exclude children from this process; they are aware of what’s happening, and there are teenagers who need to be considered too. When someone receives a diagnosis, they might often say, ‘No, I don’t want my family to know’. However, it’s important to remember that your family is your immediate support” (P06, female patient).

Additionally, a caregiver stressed the importance of considering the impact of cancer on all family members, not just primary caregivers, stating, “It’s not just the lives of the caregivers that are affected, but also those of the closest family members. There may be someone who sits quietly and doesn’t say anything, but they are still impacted” (P08, male caregiver).

### 3.5. Symptoms and Treatment Preparedness Needs

Symptom management and treatment understanding were identified as crucial components of preparedness. Participants highlighted the importance of a comprehensive education on symptoms and treatment side effects. A patient emphasized the need for clear communication from medical professionals to alleviate fears and misconceptions, stating, “When the doctor tells you, ‘You have cancer’, your immediate thought is often, ‘I’m going to die’, because that’s the first thing that comes to mind. It hurts. Some people start chemotherapy but then stop it because they experience side effects like vomiting. While we all know there are positive aspects, the doctor needs to be that supportive friend, which is often missing” (P06, female patient).

Comparably, a caregiver emphasized the importance of understanding potential symptoms and situations that may arise during treatment, stating, “This is what is going to happen to me, and these are the symptoms. These are the situations you are going to encounter. That’s how I see it” (P12, female caregiver).

### 3.6. Healthcare Provider Preparedness

Dealing with healthcare providers emerged as another critical aspect of preparedness. Participants highlighted the importance of effective communication and trust in the patient–provider relationship. One patient emphasized the need for clarity in medical communication to ensure understanding, stating, “Sometimes, when people are not familiar with a diagnosis, the communication between doctor and patient can be challenging. Doctors may use many technical terms, leaving the patient confused and thinking, ‘Wait, what does this mean?’” (P06, female patient). Another participant emphasized the importance of educating healthcare providers on addressing physical and emotional needs, stating, “Help bring it to the awareness of doctors that how a patient feels can significantly impact their right to receive proper treatment” (P25, female patient).

### 3.7. Practical and Care Preparedness

Participants discussed the practical and care-related aspects of preparedness. A patient shared a personal experience of learning to care for a family member with cancer, stating, “My little sister was diagnosed with cancer in her face, and she had to have part of her face removed. During that time, the nurses taught me how to care for her because my older sister couldn’t. I ended up becoming her nurse. It was a difficult experience” (P19, female patient). Additionally, a community partner highlighted the challenges caregivers face in navigating practical aspects of care, stating, “Sometimes the caregiver is faced with the situation and thinks, ‘What am I going to do? If something goes wrong and the patient gets an infection, it’s my fault’. It’s a delicate balance, and while it’s crucial for the patient, it’s even more so for the caregiver” (P03, female community partner).

### 3.8. Financial Preparedness Needs

Participants discussed the economic impact of cancer and the need for financial preparedness. Patients highlighted the financial burden of cancer treatment. They emphasized the importance of financial planning, stating, “I always say that cancer affects all aspects of life because it involves significant financial costs. If you don’t have the financial means, you don’t have a plan” (P06, female patient). Similarly, a caregiver discussed the costs of accessing specialized care and the challenges of managing expenses, stating, “The specialists are often located far away, like in Adjuntas (center of Puerto Rico) or San Juan (northern Puerto Rico). Traveling to San Juan is costly if you live in Peñuelas (southern Puerto Rico). And if you get there only to find out that your appointment was canceled without notice, it’s a waste of your time and resources” (P12, female caregiver).

### 3.9. Spiritual Preparedness Needs

Participants highlighted the importance of spiritual preparedness in coping with cancer. While direct quotes from caregivers, community partners, and healthcare providers were unavailable, their consent or agreement with the sentiment was noted (e.g., nods, “yes” statements). One patient emphasized the significance of faith in navigating the challenges of cancer, stating, “I think the most important part is faith” (P06; female patient).

## 4. Discussion

Cancer poses significant challenges for Hispanic individuals, particularly in terms of preparedness for the journey and cancer care [38]. This study sheds light on the complex and specific needs of the Hispanic population—the parent study aimed at conducting focus groups to develop a communication intervention for Hispanic dyads. However, secondary analysis yielded additional needs for patients with cancer and their caregivers. Our sample identified and defined preparedness and preparedness needs as encompassing psychological, emotional, educational, familial, practical, financial, and spiritual dimensions.

Participants reported the need for strategies to address emotional needs, such as providing spaces for expression and support, and addressing psychosocial needs for the dyad. After assuming their new role, caregivers may experience chronic distress, depression, and stress during the cancer journey [39]. However, this is not limited and is experienced during all the patient phases, from diagnosis to treatment completion [40]. When a caregiver’s psychosocial needs are not being met, poorer mental health can be experienced throughout the cancer journey [41]. Patients must adapt to a life-threatening diagnosis, which may lead to emotional distress, depression, and anxiety [42]. This adaptation leads patients to identify emotional needs that affect them regarding the diagnosis. The impact of the diagnosis forces them to confront issues related to their sense of self, identity, fears, and uncertainty [43]. Given the importance of addressing psychological needs and the impact on both patients and caregivers, our results are consistent with previous findings, highlighting how caregivers and health providers recognize and address these needs and the gaps in community support.

Tailored educational interventions are crucial for empowering patients, caregivers, and family members to navigate the complexities of cancer care [44]. Dyadic-aimed educational interventions that target meal support, care, symptom management, nutrition, and treatment improve the quality of life of patients and caregivers [7,44]. The results suggest that the educational aspect should be tailored to the patient’s needs. Consistent with the literature, participants present a need to understand the course of symptoms and treatment. Tailored caregiver interventions also assist in alleviating the burden and strain for caregivers [45]. This aligns with our results; participants identified the need for guidance and support for caregivers and family members regarding cancer. Moreover, psychoeducational interventions improve patients’ and caregivers’ depression, anxiety, quality of life, self-efficacy, and physical health [46]. Cancer also imposes substantial financial burdens, specifically among people with less access to healthcare [43]. Economic concerns and hardships like bill management and paying for care lead to financial stress. The patient population desires information support and education to assist in targeting these needs [47]. The socioeconomic status observed in the income of our sample highlights the potential financial challenges this population faces.

Beyond primary caregivers, family members play pivotal roles in providing support throughout the cancer journey [48], highlighting the emergence of the theme among our sample. Healthcare providers identify the role of family members on the patient’s treatment journey, where they can serve as advisors regarding treatment choices for patients; however, not addressing their needs may lead to distress, anxiety, and depression due to choices they must face and coming to terms with acceptance [49]. Identifying and addressing family member needs may also positively impact QOL and anxiety among extended family members [50]. Along the dyad, family members value clear, attentive, and genuine communication from their caregivers, as it assists in meeting the needs of the family and dyad [51]. Similar to the distribution of our sample, unmet family members’ needs are seen as higher in women than men [50].

Finally, our sample underscored spiritual beliefs’ role in the cancer journey. Spiritual beliefs and practices serve as sources of strength and comfort for individuals coping with cancer; they assist in managing the emotional and psychological challenges associated with a cancer diagnosis [52,53,54]. Moreover, religious and spiritual needs also impact treatment decisions and planning [55]. Among Hispanics, spirituality and religious engagement significantly influence how patients with cancer and caregivers cope, offering emotional support and improving their quality of life [56]. Additionally, regardless of how often they attend religious services, participating in spiritual practices and holding spiritual beliefs may impact the health and psychosocial well-being of Hispanics coping with cancer [56].

The findings of this study offer crucial insights for enhancing clinical practice, especially for Hispanic patients and their caregivers dealing with a cancer diagnosis. Healthcare providers—including physicians, nurses, and psychologists—can implement these results by incorporating culturally sensitive interventions that address the comprehensive needs identified in the study. This includes offering tailored educational programs to improve the understanding of symptoms and treatment, providing psychosocial support to manage emotional and psychological stress, and ensuring resources are available to address financial and practical challenges. Additionally, involving community members can assist healthcare providers in understanding and addressing the unique needs of Hispanic patients, facilitating better communication and trust.

Implementing these strategies can lead to enhanced communication between patients and healthcare providers, improved adherence to treatment plans, and reduced stress and anxiety among patients and caregivers. Recognizing and integrating the role of spiritual beliefs and practices into patient care is also essential, as these can significantly influence coping mechanisms and treatment decisions. Additionally, addressing the socioeconomic challenges faced by this population by providing financial counseling and support services can alleviate some of the burdens associated with cancer care. By applying these findings in clinical settings, healthcare professionals can offer more holistic and compassionate care, fostering a supportive environment that significantly benefits Hispanic patients and their families. This comprehensive approach improves the immediate quality of life and resilience of patients and caregivers and contributes to better long-term health outcomes and overall well-being.

## 5. Conclusions

This study highlights the multifaceted nature of preparedness among Hispanic caregiver–patient dyads facing cancer. Tailored interventions can enhance the resilience and well-being of individuals navigating the cancer journey by addressing the psychological, emotional, educational, familial, practical, financial, and spiritual dimensions of preparedness. Culturally sensitive approaches are essential for promoting inclusivity and maximizing the impact of supportive interventions.

## 6. Limitations and Future Directions

While this study provides valuable insights, certain limitations should be mentioned. The sample size and geographic scope may limit the generalizability of the findings. Future research should explore outcomes and evaluate the effectiveness of tailored interventions in addressing preparedness needs.

## Figures and Tables

**Table 1:** Sociodemographic characteristics.

Variables	$f\;(\%),\;\bar{x}$
Participants’ sociodemographic characteristics	
Sex	27
Female	24 (89%)
Male	3 (11%)
Age	53
Income	25,880
Marital Status	
Married	10 (37%)
Single	8 (30%)
Widow	2 (7%)
Divorced	1 (3%)
Participants’ characteristics	
Patients	8 (30%)
Caregivers	8 (30%)
Community partners	7 (26%)
Healthcare provider	4 (15%)

## Data Availability

Data can be shared upon request.
